# Factors Related to Self-Reported Distress Experienced by Physicians During Their First COVID-19 Triage Decisions

**DOI:** 10.1017/dmp.2021.170

**Published:** 2021-06-07

**Authors:** Francisca L. Chou, David Abramson, Charles DiMaggio, Christina W. Hoven, Ezra Susser, Howard F. Andrews, Stanford Chihuri, Barbara H. Lang, Megan Ryan, Daniel Herman, Ida Susser, Franco Mascayano, Guohua Li

**Affiliations:** 1 Department of Anesthesiology, Columbia University Vagelos College of Physicians and Surgeons, New York, New York, USA; 2 Program on Population Impact, Recovery and Resilience; School of Global Public Health, New York University, New York, New York, USA; 3 Departments of Surgery and Population Health Science, New York University Langone Medical Center, New York, New York, USA; 4 New York State Psychiatric Institute, New York, New York, USA; 5 Department of Epidemiology, Columbia University Mailman School of Public Health, New York, New York, USA; 6 Department of Psychiatry, Columbia University Vagelos College of Physicians and Surgeons, New York, New York, USA; 7 Department of Biostatistics, Columbia University Mailman School of Public Health, New York, New York, USA; 8 Silberman School of Social Work, City University of New York, New York, New York, USA; 9 Hunter College, City University of New York, New York, New York, USA

**Keywords:** COVID-19, health-care workforce, stress

## Abstract

**Objective:**

The aim of this study was to identify factors associated with distress experienced by physicians during their first coronavirus disease 2019 (COVID-19) triage decisions.

**Methods:**

An online survey was administered to physicians licensed in New York State.

**Results:**

Of the 164 physicians studied, 20.7% experienced severe distress during their first COVID-19 triage decisions. The mean distress score was not significantly different between physicians who received just-in-time training and those who did not (6.0 ± 2.7 vs 6.2 ± 2.8; *P* = 0.550) and between physicians who received clinical guidelines and those who did not (6.0 ± 2.9 vs 6.2 ± 2.7; *P* = 0.820). Substantially increased odds of severe distress were found in physicians who reported that their first COVID-19 triage decisions were inconsistent with their core values (adjusted odds ratio, 6.33; 95% confidence interval, 2.03-19.76) and who reported having insufficient skills and expertise (adjusted odds ratio 2.99, 95% confidence interval 0.91-9.87).

**Conclusion:**

Approximately 1 in 5 physicians in New York experienced severe distress during their first COVID-19 triage decisions. Physicians with insufficient skills and expertise, and core values misaligned to triage decisions are at heightened risk of experiencing severe distress. Just-in-time training and clinical guidelines do not appear to alleviate distress experienced by physicians during their first COVID-19 triage decisions.

The coronavirus disease 2019 (COVID-19) pandemic has brought on waves of uncertainty both within clinical settings and for the general public. During the pandemic’s initial surge, New York State experienced one of the most devastating rises to peak levels of novel coronavirus cases in the United States, characterized early on by a lack of personal protective equipment and ventilators.^1^ On March 23, Governor Andrew Cuomo ordered New York hospitals to increase their capacity by 50% to accommodate the anticipated patient surge.^2^ Clinicians, health system administrators, and public health officials began considering the need for alternative standards of care to address the problem of scarce resource allocation.^3^

Given the competing demands for intensive care unit (ICU) beds and personnel, clinicians were confronted with making triage decisions regarding ventilator use and maintenance of life-support among critically-ill COVID-19 patients. Although clinicians are trained to make triage decisions based on clinical criteria, having to make such decisions given a lack of resources is a unique stressor to most practicing physicians. As such, anxiety and distress among clinicians taking care of patients with COVID-19 became a growing concern.^4^

Sources of anxiety among clinicians with respect to patient care during this time include concerns about a lack of access to up-to-date information, lack of personal protective equipment, and the ability to provide competent care in new environments.^4,5^ In an effort to reduce the distress experienced by clinicians, experts recommended establishing guidelines and policies pertaining to the distribution of resources, such as hospital beds, medications, and ventilators.^6,7^ Receiving COVID-19-related clinical guidelines and specific COVID-19 just-in-time training (JiTT) may be helpful for reducing clinician distress. Additionally, interventions protecting clinicians’ mental health may help ensure patient safety and quality of care.^8–11^

JiTT is a method of out-of-classroom foundational instruction, at times in the form of a video or simulation, aimed at bolstering clinical learning. Previous studies have reported that JiTT is an effective tool in helping physicians-in-training improve their confidence and comfort level with procedural interventions (eg, suturing, lumbar punctures, and intraosseous needle placement),^12–14^ while also reinforcing skills that were previously learned (eg, splinting).^14^ Clinical guidelines have long been used by clinicians as a framework (informed by evidence-based medicine) for delivering patient care and developing standards of care.^15,16^ Prior research indicates that clinicians generally regard clinical guidelines positively and believe in the implementation of clinical guidelines to improve the quality of patient care.^17,18^ Currently, research on the use of clinical guidelines is largely limited to patient outcomes. The potential mental health benefit of these guidelines for clinicians remains unclear. As more clinical guidelines and protocols for the management of patients with COVID-19 are developed (both on a national and institution-specific level), an evaluation of the association between receiving these guidelines and physician distress would help to fill the gaps in clinical guideline research.

While many factors contribute to the level of distress experienced by physicians when faced with making clinical decisions, such as experience, expertise, time pressures and clinical setting,^19–23^ it remains imperative to determine if JiTT and the use of clinical guidelines (both helpful in providing physicians with more information as the COVID-19 pandemic unfolds) are associated with reduced levels of physician distress when it comes to making COVID-19-related triage decisions. Our study aims to assess factors, such as COVID-19-related JiTT and clinical guidelines, associated with distress experienced by physicians during their first COVID-19 triage decisions.

## Methods

This cross-sectional study is an analysis of the first sample of data drawn from the COVID-19 Healthcare Personnel Study (CHPS), a longitudinal study aimed at understanding and mitigating the adverse impact of the COVID-19 pandemic on healthcare workers in New York. The study was approved by the Columbia University Irving Medical Center Institutional Review Board. A waiver of written informed consent was obtained, and all participants provided informed consent electronically. The first cluster of data was collected from April 29, 2020, to May 1, 2020, through a questionnaire sent out to all licensed physicians, nurse practitioners, and physician assistants in New York State by the state health commissioner’s office. As of May 1, 2020, there was a total of 308,314 confirmed novel coronavirus cases in New York State.^24^ At that time, New York State had reached a peak number of novel coronavirus cases with hospitals operating at maximum capacity.

The baseline survey consisted of 40 questions pertaining to demographics, professional practice, living environment, interactions with COVID-19 patients, attitudes toward the COVID-19 response, personal and mental health impacts of the COVID-19 pandemic, receiving clinical guidelines and JiTT, access to ancillary assistance, and attitudes toward the future. The overall response rate of the CHPS baseline survey was approximately 10%. The present study was limited to data from physician respondents involved in treating COVID-19 patients. The exposures of primary interest were defined as whether or not respondents received COVID-19-related JiTT and clinical guidelines based on answers to the following questions: “Did you receive just-in-time training to perform your current responsibilities?” and “Have you been given formal guidelines on allocating to COVID-19 patients such resources as ventilators, beds, medications, resuscitation, or none of the above?”

The outcome measure was self-reported distress experienced by physicians during their first instance of COVID-19 triage decision-making. The survey assessed distress scores using an ordinal scale from 1 to 10, with 1 representing not at all distressed and 10 representing extremely distressed. The self-reported distress score was analyzed first as a continuous variable and then as a dichotomous variable, wherein the presence or absence of severe distress was defined as a distress score of ≥9 or ≤8. Demographic data and professional characteristics included age, gender, primary specialty, years of practice, redeployment status, having sufficient skills and expertise, and COVID-19 triage decision alignment with respondents’ core values (based on answers to the question, “Was the decision consistent with your core values?” in reference to respondents’ first COVID-19 triage decisions).

Frequency distributions of demographic and professional characteristics (ie, age, gender, primary specialty, posttraining years of practice, skills and expertise, COVID19-related redeployment, and whether triage decisions were consistent with physicians’ core values) were tabulated by JiTT and clinical guidelines status. Pearson’s chi-squared tests were used to compare JiTT, clinical guidelines status, and severe distress on demographic and professional characteristics. Student’s t- and 1-way analysis of variance (ANOVA) tests were used to assess differences in self-reported distress score by demographic and professional characteristics.

Multiple linear regression and logistic regression models were used to assess the associations of physician-reported distress scores and severe distress during physicians’ first COVID-19 triage decisions with JiTT, clinical guidelines, and demographic and professional characteristics. Statistical significance was set at *P* < 0.05 for 2-tailed tests. All data analyses were performed using IBM SPSS Statistics (Armonk, NY).

## Results

Of the 1341 physician respondents, 164 (8.4%) reported having been involved in the treatment of at least 1 COVID-19 patient. Most of the 164 physicians were 40-59 y old (female: 66 [42.3%]; male: 97 [59.9%]), specializing either in internal medicine (51 [31.1%]) or 1 of the specialties defined as other, which mostly included subspecialty fields (63 [38.4%]). Additionally, most had been practicing for 1-10 y postresidency (57 [36.5%]) and had not been redeployed from their usual clinical setting or activity (134 [82.2%]). The majority of the 164 respondents reported that their training provided them with sufficient skills and expertise for their current responsibilities (140 [85.4%]). Most also indicated that their first COVID-19 triage decision was consistent with their core values (136 [82.9%]).

Half of the respondents (82 [50%]) reported receiving JiTT. Younger age, fewer postresidency years of practice, and redeployment predicted having received JiTT, with redeployment as the most significant predictor of having received JiTT. Physicians in the 30-39 y age group were much more likely to have received JiTT than physicians aged 60 y and older (65.9% vs 36.7%, respectively; *P* = 0.021). As age also correlates to postresidency years of practice, there was a higher percentage of JiTT use among physicians who have practiced 1-10 y compared with the percentage of JiTT use among physicians who have practiced ≥31 y (37 [64.9%] vs 14 [35.9%]; *P* = 0.037). Additionally, the proportion of physicians who received JiTT in the redeployed group was greater than the proportion of physicians who received JiTT in the non-redeployed group (22 [75.9%] vs 59 [44.0%]; *P* = 0.002). Having sufficient skills and expertise was the most significant predictor of having received COVID-19 related clinical guidelines. A greater percentage of physicians who reported having sufficient skills and expertise received clinical guidelines compared with the group that reported having insufficient skills and expertise (106 [75.7%] and 13 [54.2%]; *P* = 0.029) (Table 1).

**Table 1.** Frequencies and proportions of physicians having received just-in-time training and clinical guidelines by demographic and professional characteristics among physicians who made COVID19-related triage decisions, COVID-19 Healthcare Personnel Study, New York

Characteristic^a^	Total No. (*n* = 164)	Received just-in-time training No. (%)	*P*-Value	Received clinical guidelines No. (%)	*P*-Value
Age category (y)					
30-39	41	27 (65.9)	0.021	25 (61.0)	0.082
40-59	66	35 (53.0)		53 (80.3)	
≥60	49	18 (36.7)		37 (75.5)	
Gender					
Female	65	31 (47.7)	0.542	50 (76.9)	0.413
Male	97	51 (52.6)		69 (71.1)	
Primary specialty					
Internal medicine	51	25 (49.0)	0.303	34 (66.7)	0.079
Emergency medicine	30	17 (56.7)		25 (83.3)	
Surgery/Anesthesiology	20	13 (65.0)		18 (90.0)	
Other	63	27 (42.9)		42 (66.7)	
Post-training years of practice					
1-10	57	37 (64.9)	0.037	38 (66.7)	0.566
11-20	29	13 (44.8)		23 (79.3)	
21-30	31	15 (48.4)		24 (77.4)	
≥31	39	14 (35.9)		28 (71.8)	
COVID-related redeployment					
No	134	59 (44.0)	0.002	96 (71.6)	0.645
Yes	29	22 (75.9)		22 (75.9)	
Sufficient skills or expertise					
No	24	16 (66.7)	0.077	13 (54.2)	0.029
Yes	140	66 (47.1)		106 (75.7)	
COVID-19 triage decision was consistent with core values					
No	28	14 (50.0)	1.000	20 (71.4)	0.883
Yes	136	68 (50.0)		99 (72.8)	

a There were 8 physicians with missing data on age, 2 on sex, 8 on post training years of practice, and 1 on COVID-related redeployment.

**Figure 1.**
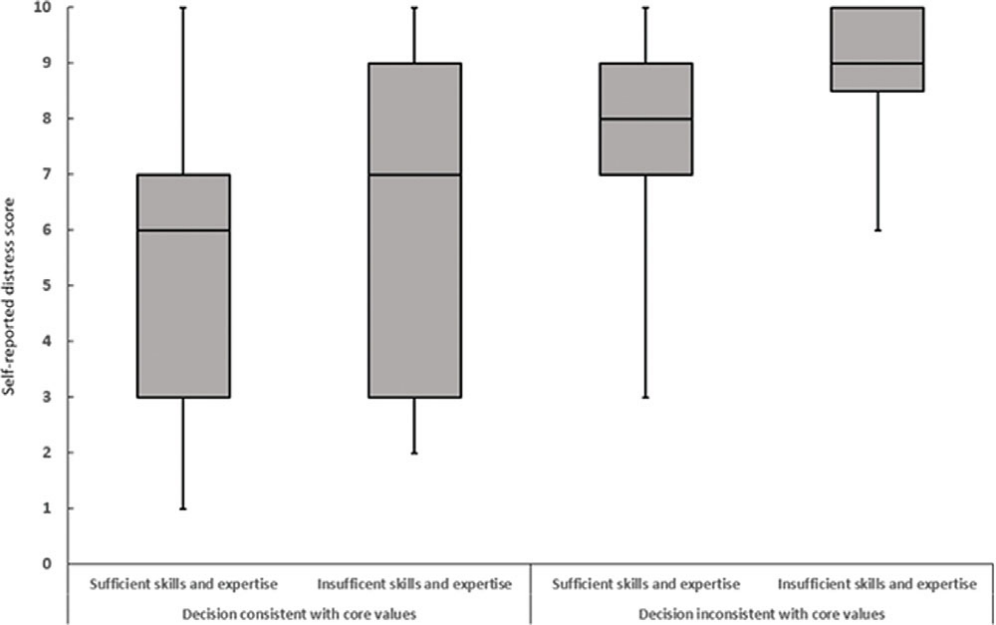
Box-and-whisker plot of physician-reported distress score during first COVID-19 triage decision according to whether the physician had sufficient skills and expertise and whether triage decision was consistent with the physician’s core values, COVID-19 Healthcare Personnel Study, New York.

The overall mean distress score among physician respondents involved in COVID-19 triage decisions was 6.1 ± 2.7. Female gender, fewer postresidency years of practice, not having sufficient skills and expertise, and COVID-19-related triage decision inconsistency with one’s core values were significantly associated with higher mean distress scores reported for physicians’ first COVID-19 triage decisions. In fact, consistency between first COVID-19 triage decisions and physicians’ core values was the most significant predictor of lower mean distress scores. Female physicians had a higher mean distress score than male physicians (6.7 ± 2.2 vs 5.7 ± 3.0; *P* = 0.01). Physicians with 1-10 postresidency years of practice had higher mean distress scores than physicians with ≥31 postresidency years of practice (6.9 ± 2.5 vs 5.6, ± 2.4; *P* = 0.045). Additionally, physicians reporting a lack of sufficient skills and expertise had a greater mean distress score than physicians who reported having sufficient skills and expertise (7.7 ± 2.6 vs 5.8 ± 2.7; *P* = 0.002). Finally, physicians who reported that their first COVID-19 triage decisions aligned with their core values had a lower mean distress score than physicians who reported that the decision did not align with their core values (5.6 ± 2.7 vs 8.4 ± 1.7; P ≤ 0.0001) (Table 2). In general, physicians who reported having insufficient skills and expertise, or an inconsistency between their first COVID-19 triage decisions and their core values, reported higher distress scores when compared with physicians who reported having sufficient skills and expertise, or consistency between their first COVID-19 triage decisions and their core values **(** Figure 1 **)**.

**Table 2.** Physician-reported distress score and prevalence of feeling severely distressed during first COVID-19 triage decision by demographic and professional characteristics, COVID-19 Healthcare Personnel Study, New York

Characteristic^a^	Mean distress score (SD)	*P*-Value	Severely distressed^b^ No. (%)	*P*-Value
Total	6.1 (2.7)		34 (20.7)	
Age category (y)				
30-39	6.9 (2.5)	0.053	11 (26.8)	0.076
40-59	5.9 (2.9)		17 (25.8)	
≥60	5.6 (2.5)		5 (10.2)	
Gender				
Female	6.7 (2.2)	0.010	15 (23.1)	0.593
Male	5.7 (3.0)		19 (19.6)	
Primary specialty				
Internal medicine	6.2 (2.7)	0.994	11 (21.6)	0.685
Emergency medicine	6.1 (2.8)		6 (20.0)	
Surgery/anesthesiology	6.1 (3.3)		6 (30.0)	
Other	6.0 (2.6)		11 (17.5)	
Post training years of practice				
1-10	6.9 (2.5)	0.045	17 (29.8)	0.072
11-20	5.6 (3.0)		6 (20.7)	
21-30	5.5 (3.1)		6 (19.4)	
≥31	5.6 (2.4)		3 (7.7)	
COVID-related redeployment				
No	6.1 (2.8)	0.794	29 (21.6)	0.597
Yes	6.2 (2.5)		5 (17.2)	
Sufficient skills or expertise				
No	7.7 (2.6)	0.002	12 (50.0)	<0.0001
Yes	5.8 (2.7)		22 (15.7)	
COVID-19 triage decision was consistent with core values				
No	8.4 (1.7)	<0.0001	16 (57.1)	<0.0001
Yes	5.6 (2.7)		18 (13.2)	
Received Just-in-Time Training				
No	6.0 (2.7)	0.550	13 (15.9)	0.123
Yes	6.2 (2.8)		21 (25.6)	
Received clinical guidelines				
No	6.0 (2.9)	0.820	10 (22.2)	0.772
Yes	6.1 (2.7)		24 (20.2)	

Abbreviation: SD, standard deviation.

a There were 8 physicians with missing data on age, 2 on sex, 8 on post training years of practice, and 1 on COVID-related redeployment.

b Severe distress defined as a score of 9 or 10 on the distress scale.

**Figure 2.**
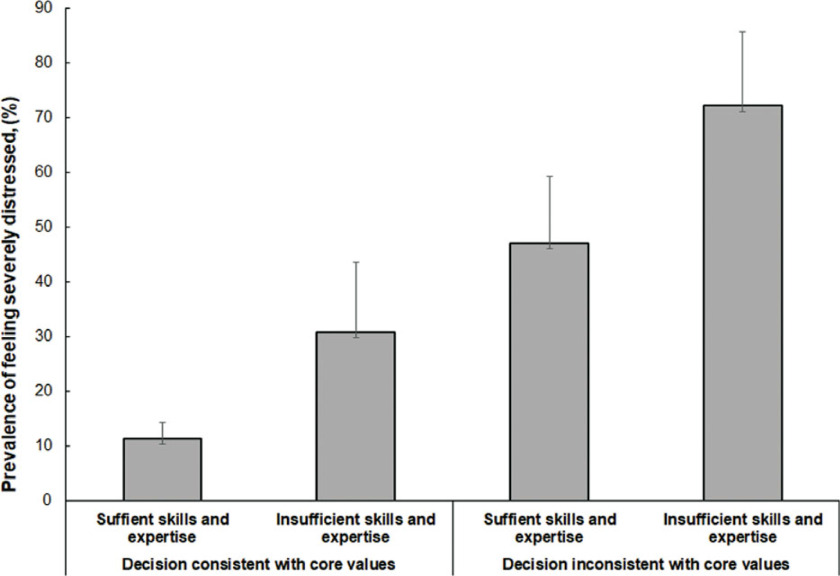
Prevalence of physician-reported severe distress during first COVID-19 triage decision according to triage decision congruity with core values and self-report of having sufficient skills and expertise, COVID-19 Healthcare Personnel Study, New York.

Overall, 34 (20.7%) respondents reported experiencing severe distress during their first COVID-19 triage decisions. Reporting insufficient skills and expertise and inconsistencies between first COVID-19 triage decisions and physicians’ core values were the most significant predictors of severe distress. More physicians reporting a lack of sufficient skills and expertise also reported having severe distress during their first COVID-19 triage decisions compared with physicians who reported having sufficient skills and expertise (12 [50%] vs 22 [15.7%]; *P* ≤ 0.0001). Additionally, physicians who reported that their first COVID-19 triage decisions did not align with their core values were more likely to have experienced severe distress than physicians who reported that the decisions aligned with their core values (16 [57.1%] vs 18 [13.2%]; P ≤ 0.0001) (Table 2). Overall, physicians who reported having insufficient skills and expertise and an inconsistency between their first COVID-19 triage decisions and their core values had a higher prevalence of experiencing severe distress compared with physicians who reported having sufficient skills and expertise and consistency between their first COVID-19 triage decisions and their core values (Figure 2).

The mean distress score for respondents who did not receive JiTT was not significantly different from the mean distress score for respondents who received JiTT (6.0 ± 2.7 vs 6.2 ± 2.8; *P* = 0.550). Similarly, the mean distress score for respondents who did not receive COVID-19-related clinical guidelines was not significantly different from the mean distress score for respondents who received clinical guidelines (6.0 ± 2.9 vs 6.2 ± 2.7; *P* = 0.820). Finally, there was no significant difference in the prevalence of severe distress between those who did and did not receive JiTT (21 [25.6%] vs 13 [15.9%]; *P* = 0.123). Experiencing severe distress also did not differ significantly among those who did and did not receive COVID-19-related clinical guidelines (24 [20.2%] vs 10 [22.2%]; *P* = 0.772) (Table 2).

When adjusted for significant covariates (age, gender, posttraining years of practice, and having sufficient skills/expertise), physicians who reported that their first COVID-19 triage decisions did not align with their core values had a mean distress score 2.3 points higher overall than that of physicians who indicated their first COVID-19 triage decisions aligned with their core values. Furthermore, physicians who reported insufficient skills and expertise had a mean distress score 1.23 points higher than that of physicians who reported having sufficient skills and expertise. The odds of severe distress for physicians who reported that their first COVID-19 triage decisions did not align with their core values was over 6 times that for those who reported that their first COVID-19 triage decisions aligned with their core values (adjusted odds ratio, 6.33; 95% confidence interval, 2.03-19.76), and the odds of severe distress for physicians who reported having insufficient skills and expertise was nearly 3 times that for those who reported having sufficient skills and expertise (adjusted odds ratio, 2.99; 95% confidence interval, 0.91-9.87) (Table 3).

**Table 3.** Associations of physician-reported distress score and feeling severely distressed during first COVID-19 triage decision with demographic and professional characteristics, COVID-19 Healthcare Personnel Study, New York

Characteristic^a^	Distress score		Severely distressed	
	β	95% CI	OR	95% CI
Age category (y)				
30-39	1.06	−1.27, 3.40	0.91	0.07, 12.80
40-59	0.89	−0.98, 2.76	1.87	0.21, 16.94
≥60	Reference	−-	1.00	Reference
Gender				
Female	0.78	−0.06, 1.62	0.97	0.37, 2.60
Male	Reference	−-	1.00	Reference
Post training years of practice				
1-10	−0.74	−3.11, 1.63		
11-20	−1.55	−3.82, 0.73		
21-30	−1.11	−2.84, 0.63		
≥31	Reference	−-	1.00	Reference
Sufficient skills or expertise				
No	1.23	0.01, 2.44	2.99	0.91, 9.87
Yes	Reference	−-	1.00	Reference
COVID-19 triage decision was consistent with core values				
No	2.30	1.11, 3.50	6.33	2.03, 19.76
Yes	Reference	−-	1.00	Reference
Received just-in-time training				
No	−0.01	−0.84, 0.81	0.83	0.32, 2.21
Yes	Reference	−-	1.00	Reference
Received clinical guidelines				
No	−0.34	−1.30, 0.62	0.99	0.31, 3.15
Yes	Reference	−-	1.00	Reference

Abbreviations: CI, confidence interval; OR, odds ratio.

a There were 8 physicians with missing data on age, 2 on sex, 8 on post training years of practice, and 1 on COVID-related redeployment.

## Discussion

Results of this study indicate that neither JiTT nor clinical guidelines had a measurable impact on self-reported distress experienced by physicians during their first COVID-19 triage decisions. Our study reveals a strong association between COVID-19 triage decision alignment with one’s core values and lower distress.

One explanation for the lack of an association between having received patient management tools (JiTT and COVID-19 clinical guidelines) and decreased levels of physician distress might be timing. If a physician’s first COVID-19 triage decision had to be made before receiving any specific and helpful decision-making tools, there would have been no way in which these tools could have reduced distress associated with the decision. Additionally, a portion of respondents indicated that, despite receiving JiTT, the training provided was insufficient. Similarly, not all respondents indicated having received multiple or all of the clinical guidelines specified in the survey (ventilators, beds, medications, and resuscitation). In fact, as of March 2020, many hospitals within the United States had not implemented robust policies and clinical guidelines pertaining to the allocation of resources (such as ventilators) needed to help manage patients with COVID-19.^25^ An insufficiency of training materials as well as a lack of clinical guidelines specific to individual patient needs may contribute to less of a role for these tools in predicting physician distress levels.

Physicians’ experience of moral distress during the COVID-19 pandemic, defined as “the experience of knowing the right thing to do while in a situation in which it is nearly impossible to do it,” is often paramount while making clinical decisions.^26^ Whether a physician’s core values pertain to the ethics involved in the allocation of scarce resources, protecting oneself and one’s family, or concerns related to limits imposed on patient care (reduced bedside interactions) among many other considerations, these core values may be challenged and lead to distress when taking care of patients with COVID-19.^26,27^ Core values are basic elements that govern personal conducts and social interactions. The distress experienced by physicians in triage decisions for critically ill COVID-19 patients arise from conflicts with personal morality rather than principles of bioethics.^28^ Even with quality patient management tools, such as clear clinical guidelines and sufficient JiTT, discordance between a physician’s core values and their ultimate clinical decisions, thereby leading to distress, may still be present. The strong association between making COVID-19-related decisions *inconsistent* with one’s core values and higher distress levels reflects a growing need for interventions focused on identifying physicians’ core values and how they may conflict with clinical decision-making. Specifically, these interventions should provide physicians with resources to combat the psychological ramifications of experiencing moral distress as a result of inconsistencies between clinical decision making and core values.^27,^ ^29,30^ Many hospital systems have developed programs, geared toward the impact of the COVID-19 pandemic, to help support both the emotional and psychological well-being of healthcare workers.^30,31^ As these programs are formed, considerations for how to mitigate the moral distress faced by healthcare workers during this time should be considered.

This study has several notable limitations. First, the sample size is small, and the respondents may not be representative of the physicians involved in the treatment of COVID-19 patients in New York State. Therefore, our findings may have limited generalizability and should be viewed as preliminary. Second, data on self-reported distress are restricted to distress experienced by physicians during their first COVID-19 triage decisions. Distress levels during these triage decisions may have reduced gradually as the COVID-19 pandemic unfolded, thus allowing clinicians an opportunity to gain more confidence in their management of patients with COVID-19. Distress associated with COVID-19 triage decisions may have also decreased with the decline of new COVID-19 cases and as more resources became available. Third, the survey did not capture data on respondents’ medical history and mental health status before the COVID-19 pandemic. An absence of these data may represent a source of bias from unmeasured confounders. Fourth, the term “core values” was not specifically defined in the survey. Therefore, respondents could have interpreted it differently, which may encompass ethical, religious, cultural, and other aspects.

Although the survey specified having respondents report on their distress during their first COVID-19 triage decisions, the term “triage” used in this context could have carried various meanings, including patient triage in the emergency department or patient triage to the intensive care unit from the floor. These distinctions would have allowed for a more nuanced analysis of the relationship between specific types of clinical guidelines and distress experienced while making decisions within the various levels of triage. Finally, associations reported in this cross-sectional study are of correlational nature and do not necessarily suggest causality. For instance, the survey data do not allow us to determine whether the respondents received COVID-19-related JiTT or clinical guidelines before making their first COVID-19 triage decision.

## Conclusion

Our study indicates that COVID-19-related JiTT and clinical guidelines do not seem to help alleviate distress experienced by physicians during their first COVID-19 triage decisions. The factor most strongly associated with decreased distress experienced by physicians during their first COVID-19 triage decisions is conformity of the triage decisions with their core values.
